# mHealth-Based Health Promotion Intervention to Improve Use of Maternity Care Services Among Women in Rural Southwestern Uganda: Iterative Development Study

**DOI:** 10.2196/29214

**Published:** 2021-11-25

**Authors:** Esther Cathyln Atukunda, Lynn T Matthews, Angella Musiimenta, Godfrey Rwambuka Mugyenyi, Samuel Mugisha, Norma C Ware, Celestino Obua, Mark J Siedner

**Affiliations:** 1 Mbarara University of Science and Technology Faculty of Medicine Mbarara City Uganda; 2 Division of Infectious Diseases School of Medicine University of Alabama at Birmingham Birmingham, AL United States; 3 Innovation Streams Limited (iStreams) Mbarara Uganda; 4 Global Health and Social Medicine Harvard Medical School Harvard University Boston, MA United States; 5 Department of Medicine and Center for Global Health Massachusetts General Hospital Boston, MA United States; 6 Harvard Medical School Harvard University Boston, MA United States

**Keywords:** mHealth app, app development, messaging, health education, health promotion, mobile phone

## Abstract

**Background:**

Antenatal care (ANC) prevents perinatal morbidity and mortality, but use of these services in Uganda remains low and maternal mortality rates are among the highest in the world. There is growing evidence that mobile health (mHealth) approaches improve timely communication of health-related information and produce positive health behavior change as well as health outcomes. However, there are limited data to guide development of such interventions in settings where ANC attendance and uptake of skilled maternity care are low.

**Objective:**

The aim of this study is to develop a novel patient-centered mHealth intervention to encourage and support women to use maternity care services in Mbarara district, southwestern Uganda.

**Methods:**

Using an iterative development approach, we conducted formative stakeholder interviews with 30 women and 5 health care providers (HCPs) to identify preferred key ANC topics and characterize the preferred messaging intervention; developed content for SMS text messaging and audio messaging with the help of 4 medical experts based on the identified topics; designed an app prototype through partnership with an mHealth development company; and pilot-tested the prototype and sought user experiences and feedback to refine the intervention through 3 sets of iterative interviews, a focus group discussion, and 5 cognitive interviews. Qualitative data were coded and analyzed using NVivo (version 12.0; QSR International).

**Results:**

Of the 75 women who completed interviews during the development of the prototype, 39 (52%) had at least a primary education and 75 (100%) had access to a mobile phone. The formative interviews identified 20 preferred perinatal health topics, ranging from native medicine use to comorbid disorders and danger signs during pregnancy. In all, 6 additional topics were identified by the interviewed HCPs, including birth preparedness, skilled delivery, male partner’s involvement, HCP interaction, immunization, and caring for the baby. Positive audio messaging and SMS text messaging content without authoritative tones was developed as characterized by the interviewed women. The postpilot iterative interviews and focus group discussion revealed a preference for customized messaging, reflecting an individual need to be *included* and *connected*. The women preferred short, concise, clear actionable messages that guided, supported, and motivated them to *keep alert* and seek professional help. Complementary weekly reminders to the women’s significant others were also preferred to encourage continuity or prompt the needed social support for care seeking.

**Conclusions:**

We used an iterative approach with diffuse stakeholders to develop a patient-centered audio messaging and SMS text messaging app designed to communicate important targeted health-related information and support rural pregnant women in southwestern Uganda. Involving both HCPs and end users in developing and formulating the mHealth intervention allowed us to tailor the intervention characteristics to the women’s preferences. Future work will address the feasibility, acceptability, and effectiveness of this design approach.

## Introduction

### Background

Antenatal care (ANC) reduces perinatal and maternal morbidity and mortality by detecting and treating prenatal complications and identifying women classified as high risk to ensure delivery in skilled settings [1-5]. ANC also provides an opportunity to support women, families, and communities at a critical time in the course of a woman’s life [3]. However, the use of perinatal services in Uganda remains low, with correspondingly high rates of unskilled home deliveries [6].

To avert maternal and perinatal deaths, the World Health Organization has called for the development and evaluation of adaptable and context-specific health solutions to promote ANC uptake, including interventions that involve delivering health care or medicine practice over mobile devices (mobile health [mHealth]) to empower women to overcome barriers to care [3]. A mechanism by which mHealth apps might affect positive health benefits is through engendering social support for the end user. Previous studies have observed a significant positive relationship among perceived social support, health care seeking, breastfeeding practices, and infant care practices among mothers [7,8]. Social support can mitigate structural and physical barriers to health and facilitate self-efficacy to complete positive health behaviors [9,10]. Several studies have found mobile phone–based messages to be motivational or inspirational or to offer a source of social support [11], cues to action [12], or a source to challenge and debunk negative beliefs [13], leading to the desired change. However, despite the successes in pilot studies elsewhere, enthusiasm from the public sector, and nearly ubiquitous availability of mobile phones in Uganda [14], mHealth interventions to motivate improved outcomes among pregnant women have not been adopted on a larger scale [15].

In all, 2 systematic reviews have identified methodological issues with prior work, including ambiguous descriptions of the interventions and their mechanisms of impact, which have generally neglected to base interventions on behavioral theory [5,16]. Others have alluded to the vital importance of the content of the message in supporting and affecting behavior change [17]. Therefore, mHealth interventions present an opportunity to address barriers to health care use through a multipronged approach by (1) teaching positive health behaviors and addressing specific health concerns (predisposing factors), (2) empowering and strengthening informed decision-making (enabling factors), and (3) improving the perceived need for use of the available services [5,16,18,19]. Others have suggested that mHealth interventions such as SMS text messaging, preloaded (or preinstalled) apps, and voice- and web-based portals may help individuals to improve eHealth literacy, internalize benefits of health services, and function as a decision-support tool at the point of care [20-22]. These interventions enhance and support healthier lifestyles, empower or enable individuals to seek help, address specific health concerns, change behavior patterns, and strengthen informed decision-making. mHealth interventions may also strengthen social relationships and support positive health behavior through SMS text messaging reminders [5,16,18,19,23].

### Objective

Despite the vast research on recommendations for optimized mHealth care, there are insufficient published data on the mHealth app development process. The development of many mHealth apps has been led by developers and investigators, with limited input from end users or with input restricted to postintervention consumer satisfaction ratings such as *like or dislike* to assess usability [24]. A participatory design process that considers clients’ and caregivers’ needs and expectations from the development phase may improve uptake and sustainability of mHealth interventions. In this study, we describe the development of a novel automated SMS text messaging and audio messaging patient-centered app designed to motivate and support women to present for ANC in rural southwestern Uganda and, ultimately, opt for skilled delivery. Our overarching aim for this paper is to describe an iterative design process usable for other groups that are designing and implementing similar interventions to promote perinatal health in low-literacy, resource-poor settings.

## Methods

### Study Design

**Figure 1:**
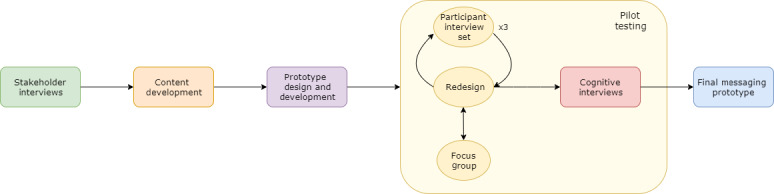
Iterative development of a novel messaging app prototype.

We used an iterative design (Figure 1) that comprised the following steps: (1) stakeholder interviews (end-user women and maternal health care providers [HCPs]) to identify key health education topics relevant to the ANC period and characterize women’s preferences for an mHealth-based, social support intervention (formative interviews with predevelopment end users); (2) content development for SMS text messaging and audio messaging with the input of 4 medical experts (2 obstetricians and 2 midwives) for the ANC topics as identified and characterized by the stakeholders; (3) design of an app prototype through partnership with an mHealth development company; and (4) pilot-testing the prototype and obtaining feedback for content refinement through (i) 3 sets of iterative exit interviews (pilot participants), (ii) a focus group discussion (FGD), and (iii) cognitive interviews to further explore user experiences and refine the updated message components and maximize potential impact and sustained use by rural pregnant women.

### Setting

#### Stakeholder Interviews

We conducted in-depth qualitative interviews with 2 groups of stakeholders: (1) target end users and (2) HCPs. A total of 40 women were invited to complete in-depth interviews, but saturation was attained after 75% (30/40) were interviewed. The women were purposively selected from rural Mbarara district, southwestern Uganda, during the period December 2018 to March 2019. These women were interviewed to identify key health education topics relevant to the ANC period as well as characteristics of a preferred mHealth app. The women were recruited from 10 villages located within 20 km of Mbarara Regional Referral Hospital (MRRH) with the help of existing village health teams (VHTs). VHTs are composed of community-based volunteers who are identified by community members and given basic training on major health programs to mobilize and sensitize communities to use available health services [25-27]. Eligible women for these predevelopment interviews included adults (1) aged ≥18 years (2) who had delivered a baby within the past 3 months, (3) owned or had access to a mobile phone, and (4) were able and willing to give informed consent. The purposeful sample was intended to represent women with differing experiences of pregnancy and ANC and included 15 women who delivered at home and 15 who delivered at a health facility.

The interviews were open-ended and organized to cover predesignated core topics. An interview guide was developed and pilot-tested using the constructs of the Healthcare Service Utilization Model as reported elsewhere [26,27] and the Technology Acceptance Model [28,29] (Multimedia Appendix 1). This open-ended approach ensured systematic coverage of specific areas of interest while allowing for unanticipated content to emerge. The interview topics included information and preferred ANC topics considered useful in supporting women during their pregnancy journey, attitude toward using mHealth technology, performance expectancy, effort expectancy, social influence, facilitating conditions, self-efficacy, anxiety, behavioral intention to use technology, and potential technology engagement or fatigue. Specific data on preferences for messaging, content, frequency, preferred language, length, and timing were also sought. A brief questionnaire at the outset of each interview was administered to collect demographic information (eg, age, occupation, and educational background).

In all, 5 key HCPs (2 obstetricians and 3 midwives) were purposively identified from MRRH and another rural maternity health center in Mbarara district. The HCPs included (1) adults aged ≥18 years (2) who were actively engaged in maternity care or policy implementation in Uganda or both, 3) had at least 5 years of experience as HCPs at a busy maternity center, and 4) were able and willing to give informed consent. They were interviewed to explore key ANC health education topics and information to inform the development of the mHealth-based app.

All interviews took place at a private location mutually agreed upon by the participant and the interviewer. Each interview lasted 50-70 minutes. Written informed consent was obtained at the outset of each interview session. Qualitative interviews were digitally recorded with the participant’s permission and transcribed verbatim.

#### Content Development

Using the formative qualitative interviews with the end users, we identified key health education topics along with ANC messages that could be developed to increase *ownership*, engagement, usability, and acceptability by the intended recipients [28,29]. The HCPs identified additional topics that were a critical part of the health education framework during ANC visits [30].

We used Behavior Change Technique Taxonomy version 1 (BCTTv1) [17,31] because it offered a reliable structure to identify, define, interpret, and characterize key components (active ingredients) of our intervention messages aimed at improving the use of maternity care services. Information was grouped within the BCTTv1 components identified as follows: (1) goal setting (outcomes: improving health-related knowledge and skilled delivery), (2) goal setting (behavior: presenting for ANC and avoiding risky behavior), (3) action planning (planning for scheduled visits, financing, actionable messages, partner involvement, and birth preparedness), (4) feedback on behavior (progress monitoring and app interaction features), (5) prompts or cues (follow-up messages or reminders and information cues, eg, danger signs during pregnancy), (6) credible source of information (systematic content development using experts and continuity of care through regular customized information), (7) instruction on how to perform the behavior, (8) information about health consequences (cautionary social and emotional consequences of, or regrets related to, poor health-seeking behavior), (9) what to do regarding, or where to seek, care or redress (problem solving), (10) review goals (interaction with HCPs and how to review progress), (11) embedded self-monitoring information on progress or preparedness, and (12) active social support for users through regular reminders (self-reminders or through identified social networks).

In all, 4 medical experts (2 obstetricians and 2 midwives), different from those interviewed, were engaged to contribute content for the first draft of the SMS text messages and audio messages, identified by the women and the HCPs, to ensure quality and consistency. The content and frequency of these messages were also based on the type of phone, network, preference, and need to ensure effectiveness and usefulness as well as avoid repetition, fatigue, and burdensomeness.

#### App Prototype Design and Development

**Figure 2:**
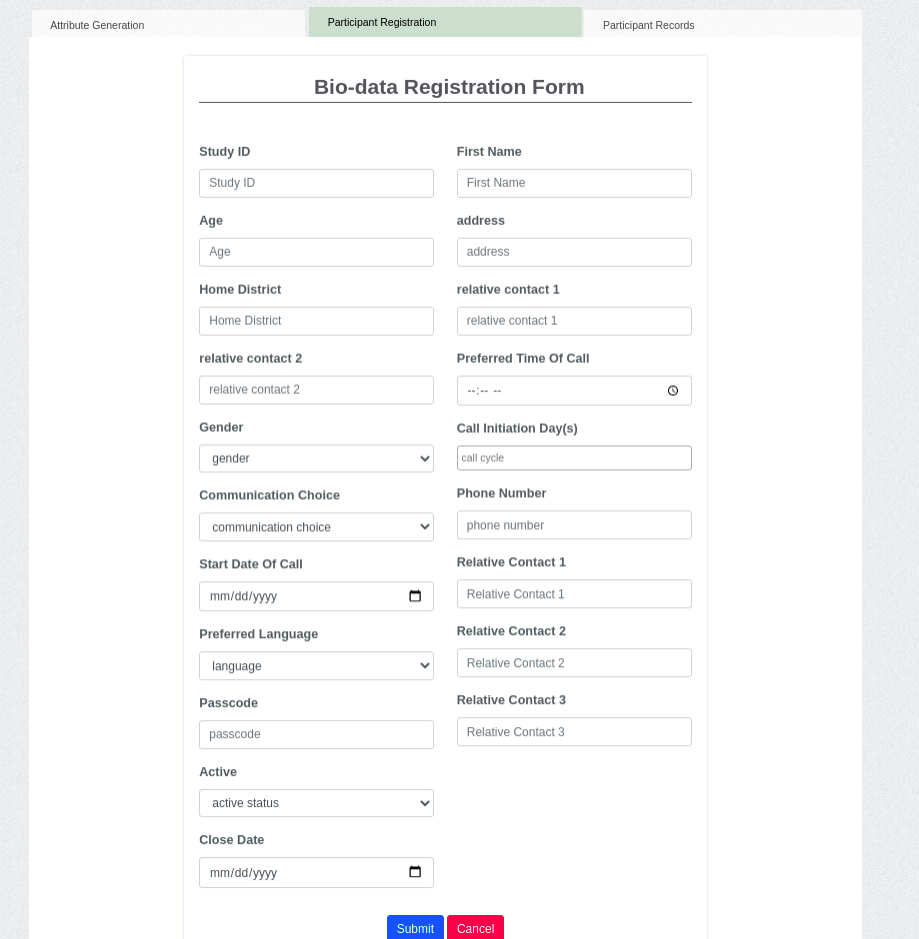
The e-Birth app biodata registration form interface.

During the intervention design, we worked with *iStreams*, an mHealth app development company in Mbarara town with an existing mHealth platform in Uganda [32]. This local developer designed an initial and novel app prototype that included both SMS text messaging and audio messaging for pilot testing. The design allows training manuals and behavior change communication materials to be integrated within a mobile app. Its unique multimedia design also allows women to listen to messages in their own language or view culturally relevant visuals (such as those that identify danger signs or complications, getting prepared for delivery, expected date of delivery, childbirth checklist, and others). Women were able to register on this platform and be tracked throughout their pregnancy and postpartum periods, receive automatic or scheduled SMS text message reminders, SMS text messages, audio messages, or notifications about upcoming appointments. In addition, the app stores medical information and allows real-time submission of data directly from a mobile phone, allowing managers and supervisors to access up-to-date data on health outcomes. The elements, content, and patterns of the SMS text messaging reminders were customized and the prototype presented as an *eBirth* platform for SMS text messaging and audio messaging (Figure 2). All messages were developed in English and then translated into the local language, Runyankole, by an experienced translator to ensure that context was maintained. Messages were dispensed in either English or Runyankole as preferred by the recipient. Fixed SMS text messaging data were stored in a secure cloud with *iStreams*, which is Health Insurance Portability and Accountability Act compliant.

We used the *Bendixen* approach [33] for designing and developing a user-centered mHealth app, considering 5 overarching goals: (1) ease of use, (2) engagement, (3) education and preparation, (4) motivation and support, and (5) tailoring the system and personalizing the information for end users. Messages were intended to communicate information on the benefits of nutrition, exercise, presenting for ANC, skilled delivery, partner involvement, birth preparedness, monitoring danger signs, and overcoming barriers to access maternity service. The topics of these messages were identified and characterized by both the women (end users) and the HCPs during the predevelopment stage, and the content was developed by health experts. Scheduled SMS text messaging reminders were incorporated as part of the intervention as a stimulus, prompt, or cue to take action.

#### Prototype Pilot Testing

Through the VHTs, we screened and enrolled 30 pregnant women (3 successive iterations of 10 pregnant women) from communities residing within 20 km of MRRH who had not presented for ANC by the beginning of their third trimester (determined by their last menstrual period) to test and assess preliminary feasibility, acceptability, and usability of the novel app through postuse qualitative interviews. An iterative approach of interviewing 10 women in each of the 3 groups was also considered sufficient to obtain rich, specific, and purposefully focused information from participants who had been exposed to the intervention [34]. These novel messages and their content were also tested to ensure ownership, relevance, consistency, and expectations among these pregnant women.

After enrollment, messages were sent to these 30 women (audio messages or SMS text messages or both), depending on the participant’s choice. Women with access to a mobile phone in their household were registered, and they received the current version of the messages through the *eBirth* app prototype (Figure 2) for at least 3 months, a period of time chosen to include a minimum of 3 ANC visits and delivery. Messages were sent in a specific sequence, depending on the month of pregnancy, to cover appropriate topics identified in the formative interviews. In the case of some women, SMS text messaging reminders were incorporated and sent to their significant others or social supporters to help remind them and support them on their upcoming ANC visit and maternity journey [23]. However, the social supporters were not given any prior recommendations or instructions guiding them on how to respond to the SMS text messaging reminders because the intervention was designed to provide social support by building on already existing supportive relationships of the study participants.

To test for frequency preferences, we first sent out messages daily for 2 weeks, then weekly at the chosen times, and then twice a week alternating between 8 AM and 8 PM. A message delivery log was monitored on the app platform. We followed the enrolled women through delivery. Upon completion of the 3-month message delivery period, we interviewed the women using semistructured questionnaires to obtain feedback on content, preferred terminologies, language, and ease of use in obtaining the needed support. We assessed phone use and responsiveness by how often the women read the SMS text messages, received calls, texted back or texted at all, confirmation of receipt, phone calls made, and the times when they missed calls or did not read the SMS text messages sent to them. The women were interviewed on technology acceptance, performance and effort expectations, whether they preferred SMS text messaging or audio messaging as a medium of information delivery, their attitude toward SMS text messaging or audio messaging technology, other preferred terminologies, content scheduling, facilitators, technology engagement, convenience, social influence, facilitating factors, anxiety, need for help using the app (self-efficacy), behavioral intention to use, and preliminary feasibility (network challenges, phone ownership, battery life, resources, frequency, and timing).

The app prototype was modified based on feedback from each iterative round of the pilot interviews. After the third modification, of the 30 participants, 10 (33%) who had had similar exposures to the intervention were randomly recruited from the pilot to constitute an FGD aimed at further refining the relevant message components and helping to limit or prioritize the number of topics included in the messaging app as recommended [35]. Finally, 5 cognitive interviews with a new set of women were conducted to further refine the updated messages and maximize potential impact and sustained use by rural pregnant women.

### Data Analysis

We described demographic and clinical data for all qualitative, iterative, and FGD interview participants using standard descriptive statistics. Qualitative analysis began with repeated review of the initial transcripts to identify relevant topics of ANC care, as well as characterization of a preferred mHealth app. Qualitative data were coded with the aid of the data management software, NVivo (version 12.0; QSR International). Coded data were iteratively reviewed and sorted to identify repeated themes (topics) arising from the data. Themes were generated using inductive content analysis [36]. The suggested content consisted of descriptive labels that defined and specified each theme (topic) meaning, along with illustrative quotes taken from the qualitative interviews. Themes were harmonized to be inclusive throughout all the development stages. Coding was guided by questions about attitude, perceived importance, usefulness, responsiveness, preliminary feasibility, experience with the messaging app, and suggested changes. Negative, positive, and neutral perceptions as well as attitudes were also identified and coded. Data analysis was performed jointly by ECA, GRM, and JN. Both JN and ECA double-coded 5 sampled transcripts, yielding a Cohen κ of 0.796. Together with GRM, we resolved disagreements until we were satisfied with the consistency in our coding to generate a codebook. We aimed at ensuring consistency in coding.

For the iterative interviews, data for frequency, timing, and frequency of messaging during the iterative testing were described using Stata software (version 12.0; StataCorp).

### Ethics Statement

All personnel involved in the project had relevant training in human subject research ethics. The study was reviewed and approved by the institutional ethics review committees of Mbarara University of Science and Technology and the Uganda National Council for Science and Technology, Kampala, Uganda. All consenting participants gave written informed consent before study enrollment; in the case of those who could not write, a thumbprint was obtained on the consent form as approved by the ethics committees.

## Results

### Stakeholder Interviews

A total of 30 women participated in the formative qualitative in-depth interviews. In addition, there were 3 iterative groups of 10 women each (30 pilot participants), 1 FGD with 10 women from the pilot, and 5 new individual cognitive interviews. Ultimately, 75 women were separately involved in the development, refining, and testing of the message content for this app. The median age of the women interviewed was 28 (IQR 24-35) years. Of the 75 women who completed interviews, 39 (52%) had at least a primary education and 75 (100%) had access to a mobile phone and previous experience with SMS text messaging or receiving a phone call. Of the 75 women, only 15 (20%) had access to an Android phone. All women were able to receive and initiate an audio call. Of the 75 women, 62 (83%) were able to read and send SMS text messages in English or Runyankole and 49 (65%) preferred both audio messaging and SMS text messaging as a medium of message delivery. Twice weekly (35/75, 47%) and weekly (34/75, 45%) messages were preferred. Of the 75 participants interviewed, 48 (64%) preferred messages sent before 8 AM and 75 (100%) preferred audio calls lasting 1-2 minutes. The rest of the demographic characteristics are presented in Table 1.

**Table 1:** Demographic characteristics of the study participants (N=75).

Characteristic		Formative interviews (n=30)	Iterative interviews (n=30)	Focus group discussion (n=10)	All participants (n=75)^a^
Age (years), median (IQR)		26 (20-33)	26 (21-34)	27 (21-34)	28 (24-35)
Education level >primary school, n (%)		12 (40)	15 (50)	4 (40)	39 (52)
Access to a mobile phone, n (%)		30 (100)	30 (100)	10 (100)	75 (100)
Experience with SMS text messaging, n (%)		30 (100)	30 (100)	10 (100)	75 (100)
Access to Android phone, n (%)		6 (20)	7 (23)	2 (20)	15 (20)
Able to receive and initiate phone call, n (%)		30 (100)	30 (100)	10 (100)	75 (100)
Able to read or send SMS in English or Runyankole, n (%)		25 (83)	25 (83)	7 (70)	62 (83)
**Preferred medium, n (%)**					
	SMS text messaging	6 (20)	4 (13)	2 (20)	13 (17)
	Audio messaging	5 (17)	4 (13)	3 (30)	13 (17)
	Both	19 (63)	22 (73)	5 (50)	49 (65)
**Preferred frequency of audio messages or SMS text messages, n (%)**					
	Daily	2 (7)	3 (10)	1 (10)	6 (8)
	Twice weekly	15 (50)	14 (47)	4 (40)	35 (47)
	Weekly	13 (43)	13 (43)	5 (50)	34 (45)
**Preferred timing of the messages, n (%)**					
	Before 8 AM	20 (67)	17 (57)	6 (60)	48 (64)
	Between 8 AM and noon	0 (0)	2 (7)	1 (10)	3 (4)
	Between noon and 8 PM	5 (17)	4 (13)	1 (10)	10 (13)
	Between 8 PM and midnight	5 (17)	7 (23)	2 (20)	14 (19)
Preferred length of audio call: 1-2 minutes, n (%)		30 (100)	30 (100)	10 (100)	75 (100)
Parity, median (IQR)		3 (2-4)	3 (2-5)	3 (2-4)	3 (2-4)
Household income ≥UGX 100,000 (US $ 27.78)/month, n (%)		16 (53)	14 (47)	4 (40)	36 (48)
Antenatal care visits (≥4), n (%)		17 (57)	18 (60)	6 (60)	48 (64)
Number of people providing support, median (IQR)		10 (5-16)	12 (6-18)	10 (4-12)	10 (6-12)
Choices for skilled delivery, n (%)		18 (60)	21 (70)	7 (70)	51 (68)

^a^Includes 5 cognitive interviews.

In addition, 5 key HCPs (2 senior obstetricians—a man aged 44 years and a woman aged 39 years—and 3 experienced female midwives aged 27, 38, and 55 years) were also purposively identified and interviewed during the formative phase. Detailed preliminary findings of these interviews have been documented elsewhere [26,27].

### Content Development

During data analysis, 34 topics were initially identified from the formative qualitative interviews conducted with the women and the HCPs. These topics included sexual health, mental health, family planning, comorbidities, nutrition, exercising, use of herbal medicine, ANC, pregnancy disorders, and normal labor. In all, 20 topics were considered, having been suggested by at least 50% (15/30) of the women interviewed, and 6 topics were specifically identified by the interviewed HCPs as a critical part of the health education framework during ANC visits as per Uganda’s national guidelines, making a total of 26 topics (Multimedia Appendix 2). These included birth preparedness, facility-based delivery, male partner’s involvement, getting to know your health worker or HCP interaction, immunization, and caring for the baby. Appropriate content for SMS text messaging and audio messaging was developed for these key health education topics and embodied the 12 identified BCTTv1 components (Multimedia Appendix 2). All messages included both prevention and promotional information. Additional data from the formative interviews on barriers to, and facilitators of, ANC attendance and skilled delivery had been already documented [26,27].

### App Prototype Design and Development

The developed SMS text messages and audio messages were uploaded onto the *eBirth* app platform in both English and Runyankole. These initial messages created the first messaging prototype. The message information could be dispensed in either English or Runyankole, depending on the recipient’s preference. A total of 26 audio messages and 26 SMS text messages were developed. Each SMS text message was restricted to between 300 and 450 characters (sent as 2-3 separate but consecutive messages, each consisting of 150 characters to avoid the splitting of messages by the various small-screen phones possessed by most of the women). Each audio message was no longer than 1 minute 30 seconds.

### Prototype Pilot Testing

#### Overview

In the qualitative interviews, 5 themes emerged related to the perceived intentions or goal of the app: attitude regarding content, wording, format, and delivery; language; frequency; length; and timing. The women identified 5 overarching app features that would maximize its impact and intended benefits. The app needed to provide (1) education and preparation, (2) motivation, encouragement, and support, (3) customization and connection, (4) easy-to-use interface, and (5) engagement and empowerment.

#### Education and Preparation

All participants interviewed during the pilot and the FGD indicated that the SMS text messages and audio messages received directly on their phones were beneficial, especially for first-time mothers-to-be or other inexperienced women who needed to understand and learn important information about pregnancy and childbirth. The women indicated that these messages, which had been developed with the help of a trusted and informed source, could reinforce the truth amid the limited and divergent information obtained from peers or relatives in their communities. According to these women, this timely information could support them to confidently debunk misinformation about pregnancy and childbirth over time. A pregnant participant aged 21 years stated as follows:

We don’t get such accurate information from anywhere other than what we usually hear from people around us as we grow up.s..like every pregnancy is the same, or like it’s normal or like you don’t need to go to hospital which can be a big problem...with these messages coming from a real midwife, you get to learn a lot about pregnancy and childbirth and add on the little information you know and it’s very good.

Most women stated that twice-weekly or weekly SMS text messages or audio messages were sufficient to communicate, educate, and prepare them through their ANC journey. Some preferred to receive messages at approximately 7 AM so as to be able to read them at the start of the day and prepare mentally during the day. Others preferred SMS text messages or audio messages to be delivered to them at approximately 8 PM when they were done with the day’s busy schedules that could have distracted them and made them forget to read, internalize, share, or discuss the messages with their significant others in real time. The women thought that this timing could offer them ample time to learn and discuss health matters with their significant others and plan the next course of action appropriately. An addition of a single midweek SMS text message reminder to the app was preferred to support their near-monthly scheduled ANC visits. The women suggested that additional SMS text message reminders sent at least once a week to their significant others (social networks) could help them to initiate or ease into a discussion on the challenges they faced and the need to go to hospital. It was indicated that stimulation of these discussions could help the women to involve their significant others in preparation for, and mobilization of, the help and support they needed for timely access to professional care during the antenatal period. A pregnant participant aged 31 years stated as follows:

I get so busy with work during the day and it’s in the evening when I catch a break and read the messages very well...my husband is even around by that time so I can walk up to him and we talk about my needs together on how to like go see a doctor together just in case, so it’s good that way.

A postpartum participant aged 28 years added the following:

May be once weekly early in the morning. The messages are great at the start or at the end of each week...I would love it if my mother-in-law also gets the message early like during the day so that in case I get a problem, she already knows my situation and can help me to go to hospital quickly since I do not stay with my husband all the time.

#### Motivation, Encouragement, and Support

The iterative interviews indicated that the women preferred messages that were both cautionary and encouraging. For example, some women suggested delivery of messages pertaining to the danger signs of a complicated pregnancy, consequences of not presenting for ANC, or incompatible sociocultural beliefs as some of the cautionary information that motivated them to actively examine themselves after reading each message. Other women indicated that cautionary or warning-based messages *sounded surreal*, had a lingering effect, and often *forced* them to quickly seek professional care as planned or as soon as these danger signs were noticed. A postpartum participant aged 34 years stated as follows:

These messages sound so surreal but, at the same time, force you to watch out in case you are not feeling well. They can wake you up and force you to go to hospital...I tried by all means to check myself all the time I received them to make sure my pain during this pregnancy was not in vain.

All the women preferred positive messages whose tone was not authoritative and that offered encouragement. Negative, rebuking messages were said to be discouraging, redundant, annoying, and emotionally draining, especially if the recipients found themselves in a situation where they had no physical or financial support to help them take action. All the women also preferred messages that were expressed in a friendly tone*.* A pregnant participant aged 25 years stated as follows:

That call leaves a lingering memory and a lasting impression on me from the lady who talks to me on phone, it’s as if listening to someone I’m familiar with already...I mean, no one hates a good message that motivates them to do good. It’s your life alright, but you need someone to encourage you to keep going like doing the right thing, you know, so you look forward to getting another one and learn from it. It’s exciting.

The iterative interviews indicated that the women felt that the regular, continual information was a source of comfort and emotional support, which was provided by people they perceived as someone who cared about their well-being. Some women referred to the messaging app as a reliable “pregnancy companion” that helped them “navigate through their pregnancy.” Other women reported that the SMS text messages and audio messages gave them a sense of excitement and confidence knowing that “someone is watching over” them as they prepared to receive a new baby. An expectant participant aged 18 years stated as follows:

I get to hear encouraging messages and compare with how I feel there and then from someone who clearly understands these things and is also willing to navigate through this pregnancy journey with me...with these messages coming through every week, we know someone is watching over us and we always look forward to the next message.

A postpartum participant aged 30 years added the following:

These messages made me feel as if someone out there cared about me and my baby. Anyone would really love that because it’s like you have a pregnancy companion who knows you so well and moves with you along this difficult journey. Deep down you know you will make it so you are encouraged.

#### Customization and Connection

Customized reminders or SMS text messages that delivered a caring message were preferred to plain default messages, reflecting users’ need to be *included*, connected, and related to the program as desired. Participants reported that the addition of SMS text message reminders improved their attitude toward formal care by providing a responsive and caring *connection* to the health system. A postpartum participant aged 28 years stated as follows:

A message like, hi XX [name], your life is very important to us, we are reminding you to go for your scheduled ANC visit in time to avoid problems during your pregnancy and childbirth...It shows someone cares. It’s short but such a message makes you feel good and connected to your midwife.

Most women revealed a preference for audio messages whenever possible because they provided a clearer flow of information or instructions at one go. However, many women indicated that they missed some audio messages and were unable to retrieve them, unless they called back, which would cost them. However, the system also provided them an interactive feedback mechanism that participants could use to request the caller to repeat the current or previous messages and listen in as long as they desired using a designated number and a numeric key. The interviews revealed that missed calls tended to occur when women encountered unforeseeable events such as parties or burials where they could not answer their phones for fear of embarrassment or when their phones were in silent mode. Other occurrences included dead batteries, lost phones, and poor or no network, which affected the delivery of messages. However, unlike audio calls, SMS text messages could be delayed but eventually came through once the participants charged their phones, revived an active SIM card, or entered an area with network coverage. To resolve this, the women suggested both audio- and SMS text message–based delivery media, a call-in number to report such challenges, an option to provide an alternative SIM card number with better network, and an option to provide contacts of people within their social network who could agree to receive messages on their behalf or call them to the phone whenever they are nearby. The component of social networks was further expressed as important for improving the continuity of such messaging interventions in this setting. A postpartum participant aged 21 years observed as follows:

One can get both calls and SMS, or get a number to call you in case their phone is stolen, has problems or something else...we can even give you other numbers of the people we live with at home or nearby who can reach us and deliver the messages in case anything happens. That way, we can stay connected.

An expectant participant aged 35 years added the following:

It’s good because I can call back in case I missed the call or make it repeat as many times if I did not understand anything...although I am charged some money to call in, I am sure I have got the right information and support whenever I need it.

A strong interest was expressed by the women, especially those who owned Android phones, to be able to connect and interact with other pregnant women and with their HCPs. According to these women, such a platform could help them share their ongoing challenges and lean on, and get support from, their providers or colleagues who have had the same experiences in real time. A postpartum participant aged 26 years stated as follows:

For example, I could see blood for example and be lazy to go to hospital for checkup or something, and women out there on the platform could share their lived experience and push you to go quickly...or like send you alternative contact of a midwife near you, such kind of thing. I think it’s very good to get people in your condition to connect and interact with all the time.

#### Easy-to-Use Interface

All the women reported that they were able to receive SMS text messages or audio calls easily. When the app was modified to offer different choices of audio messages or SMS text messages to fit individual needs, SMS text messaging was mostly preferred by women who could read and write. However, some participants thought that SMS text messages became redundant over time and instead preferred phone calls. The women observed that SMS text messaging was ideal for delivering short, concise, actionable information; therefore, a good, relevant and comprehensive message required to be split into 2-3 SMS text messages, each restricted to 150 characters, to maintain the richness of the intended information. However, the women reported that these messages sometimes came through in the wrong sequence because of network issues. Women who were unable to read preferred audio calls as an easier option because they cover the same information clearly in less than 1 minute. However, the women were also able to share SMS text messages with other people in their social networks who could read and interpret messages for them, a possible indicator that both options could be considered to reinforce information transfer and use among women in similar settings. An expectant participant aged 19 years stated as follows:

I love calls a lot because you get to listen from a real person clearly and it feels real...A phone call is also easy to receive and just listen quickly in your local language...I always take the [SMS] message to my husband or friends to read for me and so it’s also good because they explain to me everything...but I get lazy and they get boring like after some time and confusing like if they are so long and one part [of the SMS] doesn’t come properly or is missing...but a phone call is always clear, is brief and is hard to ignore since someone takes their time to think about you and call to encourage you.

#### Engagement and Empowerment

The way the message content was delivered was very important to the end users and, as such, enabled the women to continuously engage with the program. They described a need for message content that emotionally connected them to the messenger. The message content delivered by the *great messenger* was viewed as liberating and empowering because of its ability to fulfil an existing need and desire for alternative and accurate information received directly on their personal or accessible phones. As such, SMS text messages were often stored and reviewed not only as future reference material for themselves, but also for peers who needed support and with whom they shared this material. Some women were enthusiastic about audio messages, which were thought to provide more clarity, enabling them to understand and internalize the messages faster and better. Other women preferred both. The women indicated that the delivery of clear messages was important in improving understanding, engagement, and encouraging prolonged use. For example, a postpartum participant aged 31 years stated as follows:

The caller is very good. She’s very clear in explaining things, she makes you understand everything, I mean such words like aka-TV [ultrasound scan]...she explains many issues in such a short time so well, and you can press and listen again or call for more clarification if you want...you get to learn a lot and teach others...It’s encouraging to know that she understands us very well and I like her a lot.

A postpartum participant aged 24 years added the following:

It is very liberating to know you have this information from your musawo [health worker] sent directly on your phone at all time. I can keep the messages, read them again and can share them with my friends as much as they need it. I like it a lot.

The arrival of messages was seen as an ongoing reminder to stay alert and keep examining themselves, and this had the potential to help women to respond and make better choices as and when necessary. An expectant participant aged 27 years stated as follows:

These messages help a lot and keep you alert and informed. Every message is like a reminder for me to immediately check on a few things, like how I feel and see how I am doing right then and like quickly decide on what to do in case I am in trouble.

This information and feedback was used to modify the audio messaging and SMS text messaging app. The final messages made up a message bank for future evaluation to assess feasibility, acceptability, and preliminary efficacy to influence the initiation and use of maternity care services.

## Discussion

### Principal Findings

We developed a novel patient-centered SMS text messaging and audio messaging app to support women to use maternity care services in rural southwest Uganda. We found that involving end users, including providers and health care users, in developing and formulating a messaging intervention gave the women a sense of *ownership* and inclusiveness in the app development process. The women identified 5 overarching app features. The app needed to provide education and preparation; motivation, encouragement, and support; customization and connection; easy-to-use interface; and engagement and empowerment. We therefore designed and developed a novel patient-centered and customized SMS text messaging and audio messaging app to engage rural women in southwestern Uganda and communicate important targeted health-related information to support them during pregnancy. Our pilot data support a potential role for messaging apps as a complementary approach to face-to-face health education encounters, especially when the apps are well packaged and tailored to suit the end users’ needs and preferences. We now plan to evaluate the intervention formally in a randomized clinical trial in the next phase of the app development process.

Prior studies have reported improved acceptability and sustainability of mHealth interventions when the apps involved individual participation and also involved end users in the initial stages of the design process [22,24,37-39]. Other scholars have documented the beneficial effect of customizing the app to improve engagement and education in the general population [33]. In these studies, individual needs, goals, expectations, intentions, and preferences needed to be addressed to enable active engagement with the app. Our app was developed with the involvement of multiple stakeholders, including end users, HCPs, and health and technology experts. Our analysis reinforces these findings, particularly among women who expressed a great desire for relevant and customized mHealth apps and content that educates, prepares, motivates, encourages, and supports them during pregnancy. We developed customized messages tailored to these end-user preferences for this intervention and opted for a standardized automated messaging program to ensure delivery of regular health information directly to users’ phones. This approach helped women to stay connected and motivated, and they would refer to the intervention as a “pregnancy companion.”

We used an iterative approach to develop, test, and deliver a user-driven app that was considered appropriate for a largely non-Android user community to improve utility, usability, desirability, and uptake. This was in line with previous studies that found SMS text messaging language, medium of message delivery, experience with similar technology, phone type, and characteristics to be critical in designing and delivering a culturally appropriate mHealth program that is consistent with the way people are already using the promoted technology [37,39]. The creation of an intervention that was compatible with local mobile phone types and the provision of varying delivery media for both literate and illiterate individuals to receive or access credible information support from the system, keep it for future reference, or share it with family and significant others directly on their regular phones seemed to shape the users’ perception of the intervention’s usefulness. We will next assess the impact of this iterative design approach through a planned randomized clinical trial and, if successful, through programmatic implementation to evaluate its influence on health outcomes and adoption in practice.

The end users identified key technical and design preferences and challenges of the mHealth app related to the timing and frequency of messaging, unreliable phone batteries, network issues, and lost phones. The women often chose weekly SMS text message reminders, possibly because scheduled ANC visits are near-monthly, and 2 promotional or cautionary messages at the beginning and end of each week as sufficient without causing unnecessary burden. According to previous scholars, this establishment of different preferences regarding timing and frequency helps to avoid unnecessary repetition, technology fatigue, and boredom, making the messaging intervention an acceptable tool to deliver health promotion content [40]. Shaw et al [40], however, observed that sending messages at the same time of the day could reduce the value that participants accord the messages and reduce the frequency of responses. The timing of messages at the start and end of the day were therefore varied over the course of the program but fixed at the beginning and end of the week as preferred to mitigate network and battery issues. The women in our pilot interviews additionally suggested providing an option to register both numbers of a dual SIM card mobile phone or to provide other contact numbers in their social networks, with consent, to receive reminders and messages as an alternative means to improve network connectivity, continuity, and reliability, as well as minimize the issue of missed messages because of network, phone, and battery issues apparent in this setting. In line with previous studies [23], the involvement of significant others in the pilot seemed to facilitate messaging continuity and encourage women to initiate important health discussions, enabling mobilization of needed resources and support for timely access to maternity care.

Our study included a number of strengths. We involved both providers and health care users in developing a user-friendly, culture-consistent, patient-centered automated SMS text messaging and audio messaging app to stimulate, encourage, and support rural women in southwestern Uganda to use maternity care services. This study also documents women’s expectations, experiences, perceptions, and choices of an mHealth-based technology that would benefit and support them in their local communities, subject to the standard limitations of network challenges as well as mobile phone ownership and type in the region. We developed a total of 26 audio messages (based on the 26 identified ANC topics), delivered in messages restricted to 150 characters per message to suit the different phone types accessed by women in this community. This stepped and multidisciplinary approach can inform the design and implementation of other novel patient-centered interventions that aim to reinforce *ownership*, engagement, inclusiveness, and uptake in a program operating in local communities. Our study also demonstrates a potential approach that can be used to complement face-to-face education encounters by communicating important targeted health-related information that is beneficial and offers informational and emotional social support to rural women through a novel automated audio messaging and SMS text messaging app. We used well-established conceptual frameworks to characterize and develop message content for this app, helping to make the findings more grounded, acceptable, meaningful, and generalizable.

This study also included some limitations. Our approach of using conceptual frameworks may have limited considerations of key variables, which may have influenced our direction and content of interest. There is therefore a need to assess how these specific variables differ under different settings or circumstances. Most of the people in our study setting are from a less affluent or less educated background, and they are not smartphone users, which imposes limits on internet access despite improved internet penetration through local mobile phone companies. As such, the messaging content and delivery medium were developed to suit current phone access and characteristics in similar settings and thus might not be generalizable to other settings with higher literacy or smartphone use. We were not able to develop a platform to enable direct and automated feedback and affirmation on self-goal attainment targets, an approach likely to motivate long-term use [33]. However, the ability of our app to engage with social networks and HCPs was anticipated to help women continuously share their experiences concerning their milestones, challenges, and goal attainments. Sharing such successes for others to acknowledge and learn from their own accomplishments has been documented to exceptionally motivate app users [33]. We did not assess for feasibility and acceptability of the messages in this particular work. The final version of the app is currently undergoing a pilot clinical trial to document feasibility, acceptability, and preliminary efficacy.

### Conclusions

Our study describes a process for developing and testing a novel user-friendly, patient-centered mHealth-based messaging app suitable for rural women in southwestern Uganda. We have demonstrated an iterative approach with diffuse stakeholders to develop a customized, automated, patient-centered audio messaging and SMS text messaging app designed to communicate important targeted health-related information and support rural pregnant women in southwestern Uganda. Involving both HCPs and end users in developing and formulating the mHealth intervention allowed us to tailor the intervention characteristics to the women’s preferences, thus giving them a sense of ownership and inclusiveness in the program. This approach supports a potential role for messaging apps as a complementary approach to face-to-face health education encounters. Our next step is to evaluate the intervention in a pilot clinical trial to assess its larger-scale feasibility, acceptability, and ability to affect health outcomes.

## Appendix

Multimedia Appendix 1 Interview guides.

Multimedia Appendix 2 Message content.

## Abbreviations

ANC: antenatal care
BCTTv1: Behavior Change Technique Taxonomy version 1
FGD: focus group discussion
HCP: health care provider
mHealth: mobile health
MRRH: Mbarara Regional Referral Hospital
VHT: village health team
