# Supplementary material for: mHealth-Based Health Promotion Intervention to Improve Use of Maternity Care Services Among Women in Rural Southwestern Uganda: Iterative Development Study
Source: JMIR Form Res. 2021 Nov 25;5(11):e29214. doi: 10.2196/29214 (PMC8663630; doi:10.2196/29214)
Supplement: Multimedia Appendix 1 [file formative_v5i11e29214_app1.pdf]

Participant Short Questionnaire

Participant ID __ __ __	Date __ __/__ __/__ __ __ __
Staff initials __ __ __	

Participant Demographics

Instructions: To be completed by a study RA at enrollment.

1. Date of birth: __ __/__ __/__ __ __ __
d d m m y y y y

2. Gender: ☐ Male ☐ Female

3. Marital status
☐ Married ☐ cohabiting ☐ single

4. Parity: --------------------

5. How many people stay in your household (family size)

6. How many of these are less than 18 years of age?.......................................

7. Distance to nearest health facility that

a) Offers ANC--------------------------------

b) Offers EMOC/skilled deliveries---------------------------------

8. Is this facility in 7 above free of charge?

9. How much did you spend on average for

a). each of the ANC visits --

b). last delivery---------------------------------------

10. Highest level of education achieved:

☐ None ☐ P1-P7 ☐ >P7

11. Literacy

	English	Runyankole
Able to speak	☐Yes ☐No	☐Yes ☐No
Able to read (*refer to language card*)	☐Yes ☐No	☐Yes ☐No

12. For how many years have you used a cell phone? ______

13. In your typical daily routine, approximately how often do you send SMS messages?
☐ Never ☐ Monthly or less ☐ Weekly ☐ Daily ☐ > 1/day

14. In your typical daily routine, approximately how often do you receive SMS text messages?

☐ Never ☐ Monthly or less ☐ Weekly ☐ Daily ☐ > 1/day

		SIM registration verified
**15.** Preferred cell phone number	___ ___ ___ ___ ___ ___ ___ ___ ___ ___	☐
**16.** Alternate cell phone numbers<br>(check here if none ☐)	___ ___ ___ ___ ___ ___ ___ ___ ___ ___<br>___ ___ ___ ___ ___ ___ ___ ___ ___ ___	☐<br>☐

17. Language preference for SMS: ☐ Runyankole ☐ English

18. Preferred time for SMS: __ __:__ __ (use 24 hour clock)

19. Preferred day for weekly SMS:

☐ Sunday ☐ Monday ☐ Tuesday ☐ Wednesday ☐ Thursday

☐ Friday ☐ Saturday

20. Preferred content for the SMS (max 140 characters). ………………………..
……………………………………………………………………

21. Do you yourself have a regular income? By this I mean fixed wages or a fixed salary that you receive on a regular basis, for example every two weeks or every month, and is not dependent on the seasons or on how much you work.
☐ Yes ☐ No

22. What is your estimated monthly household income
--------------------------- (UGX)

23. **Food security:** The following questions ask about what you typically eat. Please note that while this research will be used to guide future programs and policies, we will NOT be distributing food items during or after this research project. Please answer these questions as truthfully as possible so that we can accurately assess food availability in this area. Whenever we say "lack of resources", we mean not having money, land, or manpower. "Household" means all of the people who stay in the same house and eat meals together.

For each of the following questions, consider what has happened in the past 30 days. Please answer whether this happened never; rarely, meaning once or twice; sometimes, meaning 3-10 times; or often, meaning more than 10 times in the past 30 days.

	Never	Rarely	Sometimes	Often
1. Did you worry that your household would not have enough food?	☐	☐	☐	☐
2. Were you or any household member not able to eat the kinds of foods you preferred because of a lack of resources? Foods you preferred might include meat, poultry, eggs, fish, milk, matooke, spaghetti and karo.	☐	☐	☐	☐
3. Did you or any household member eat just a few kinds of food, such as only matooke or posho with either beans, groundnuts or dodo, day after day due to a lack of resources?	☐	☐	☐	☐
4. Did you or any household member eat food that you preferred not to eat because of a lack of resources to obtain other types of food? A food you preferred not to eat might include cassava, matooke flour, posho, embile, dodo, katunkuma, and entura.	☐	☐	☐	☐
5. Did you or any household member have to eat less at any meal than you felt you needed to eat because there was not enough food?	☐	☐	☐	☐
6. Did you or any household member eat fewer meals in a day because there was not enough food?	☐	☐	☐	☐
7. Was there ever no food at all in your household granaries or gardens because there were not resources to get more?	☐	☐	☐	☐
8. Did you or any household member go to sleep at night hungry because there was not enough food?	☐	☐	☐	☐
9. Did you or any household member go a whole day without eating anything because there was not enough food?	☐	☐	☐	☐

24. At any time during the past year did you or your family receive food aid? By food aid I mean free food that you got from church, clinic, non-governmental organizations, or governmental organizations? I don't mean food that you got from family or friends.

☐ Yes ☐ No

25. Where did you get food aid from? Mark all that apply:

☐ Church ☐ Non-governmental organization

☐ Clinic ☐ Governmental organization

☐ Other (specify): ____

26. Did you or your family receive food aid over the past 30 days?

☐ Yes ☐ No

27. If yes, Over the past 30 days, how many days did you eat free food that you got from church, clinic, non-governmental organizations, or governmental organizations? I

don't mean food that you got from family or friends. __ __ # of days

28. Alcohol use: In the past year, how often did you have a drink containing alcohol?

☐ Never (skip to question 9) ☐ Less than monthly ☐ Monthly

☐ 2 to 4 times a month 2 to 3 times a week ☐ 4 or more times a week

29. In the past year, how many drinks of any kind containing alcohol did you have on a typical day when you were drinking?

☐ 1 or 2 ☐ 3 or 4 ☐ 5 or 6 ☐ 7, 8, or 9 ☐ 10 or more

☐ Cannot estimate because of use of non-standardized non-bottled home-brewed Beverages

30. Social support: I am going to ask you some questions about your social support. Thinking about your current situation, for each question you may answer, 'as much as I would like', 'less than I would like', 'much less than I would like', or 'never'. I will then ask you for the number of people who provide you with each type of support.

	As much as I would like	Less than I would like	Much less than I would like	Never	# People providing this support	Spouse provides this support
1. You get visits from friends and relatives	☐	☐	☐	☐	☐	
2. You get useful advice about important things in your life	☐	☐	☐	☐	☐	
3. You get chances to talk to someone about problems at work or with your housework	☐	☐	☐	☐	☐	
4. You get chances to talk to someone you trust about your personal and family problems	☐	☐	☐	☐	☐	
5. You have people who care what happens to you	☐	☐	☐	☐	☐	
6. You get love and affection	☐	☐	☐	☐	☐	
7. You get help with household-related work	☐	☐	☐	☐	☐	
8. You get help with money in an emergency	☐	☐	☐	☐	☐	
9. You get help when you need transportation	☐	☐	☐	☐	☐	
10. You get help when I you are sick	☐	☐	☐	☐	☐	

11. In total, how many different people provide you with any of the above social support?

12. Are you a member of community support group, including church groups? ☐ Yes ☐ No

<u>Interview guide (formative interviews)</u>

Predisposing factors

1. How are you? How is your family?
2. Please tell me about your experience with the last pregnancy (probe about ANC attendance, reasons for attendance/nonattendance, facilitators/barriers to attendance, each visit at a time)
3. How was your child birth (probe hospital delivery, reasons for choice of place of delivery)
4. Tell me about the last time you attended ANC (may not be last pregnancy)
5. Please share with me your experience attending ANC (including travel, waiting time, how do they get to remember their scheduled visits)
6. How did you benefit from the ANC visits? Please give me detailed examples
7. If never attended, what happened? (probe barriers eg transport, time to health facility, availability of services, health worker attitude, knowledge gap, perceived benefits/risks, etc)
8. Can you share with me about what you generally know/think about ANC?
9. How do you think ANC is important?

Enabling factors

10. What help did you get during pregnancy, child birth (probe any social support, from whom, what kind of support, etc?
11. How do you feel about the services that were rendered to you during pregnancy/child birth
    a) by spouse
    b) by health care providers
    c) any others
12. What information was given/offered to you during pregnancy/child birth? (probe sources, usefulness, etc)
13. How did this influence your choice of place of delivery/ ANC attendance?
14. What information do you wish you received during pregnancy/child birth? (probe sources, usefulness/ perceived benefits, etc) (Note; you can refer to a list of generic ANC topics to probe
15. How would you like such information to be delivered to you? (probe mobile phone options, physical, written fliers, TV, SMS, audio calls, etc)
16. *How has been your relationship with your spouse/sexual partners/family been in your last 1 year? (Probe for any changes, support, involvement, needs to engage partner)*
17. What does your spouse think about ANC / delivery in a hospital/ delivery at home? Does he support you? How? Tell me about a time when your husband helped you to attend ANC/ go to hospital for health reasons? (*Probe about a time when her spouse's reminder/support resulted in attendance of ANC/hospital appointment*?
18. What else helps you consistently and effectively attend your ANC/ hospital appointments? *Probe about social support, other commitment strategies used.*
19. Is there anything that you would like your spouse/sexual partner/family to do to support you more during pregnancy/child birth? he doesn't (they don't) currently do? What is it? *Probe for various kinds of support – financial, physical, emotional, etc.*

Need-based factors

20. What is your opinion about child birth in health facility vs home delivery?
21. What is your perception about health care providers towards supporting your health?
22. Please explain to me how support from health workers was helpful/ discouraging
23. How is your health? (probe about perceptions of current health/health status)
24. (*if already delivered*) How did your current health influence your choice of place of delivery, ANC attendance?

25. Can you share with me the details of your experience giving birth? (Probe when they realized they were due, what happened, what they did /whom they called first, time of arrival, why if early or late arrival, probe preparedness, what prompted them to seek care, etc)
26. Can you share with me the details of your experience while pregnant (probe perceived health threats/risks, co-morbidities, need for specific information and advice?)
27. Why did you choose to deliver at this particular place? (probe perceptions about hospital/home delivery, perceived benefits of this particular place of delivery, available needs, birth preparedness)
28. *Have you ever had, a) good/bad delivery/ANC visit experience b) difficult delivery/ fetal death, miscarriage/or any other postpartum complication? What happened? What do you think you needed to avoid it? What specific information did you need?*
29. Do you have any preferred ANC topics that you think would be useful in supporting you during your pregnancy journey? (*Probe examples and detailed examples, experiences*)
30. *How did this experience change your perception about your health? (probe about perceived risks/threats, cues to action, awareness*

31. TECHNOLOGY ACCEPTANCE (formative interviews, pilot interviews and FGDs)

A. Performance expectancy:

- How useful do you think mHealth technology (probe telephone calls, SMS, reminders, preferences, convenience) can be during pregnancy (probe reminders, social support, information transfer, credible source etc)
- What would you like/expect from such an SMS/audio-based technology during your pregnancy journey (*Probe specific information on motivation to attend ANC, other motivation, increased involvement, professional knowledge/information, intention, goals, problem-solving, social support involvement, prompts/cues, instruction on how to perform, etc*
- *How would you prefer the content of any of these messages? (Probe desired tone, type of content, intention of message, goal, intended support, cues, instruction/direction on behavior)*
- How useful is this kind of technology/information for you during child birth?
- How would you like it? (*Probe for specific data on preferred language, length, timing, and interest in receiving SMS support messages,)*

B. Effort expectancy: How is it for you to use these SMS/messaging/calls? (probe skills/knowledge needed to read/understand SMS, receive/understand calls, operate phone, app interaction, feedback on behavior options, self-monitoring, etc)

C. Attitude towards technology

- How do you find it using SMS/audio calls to supplement usual information from health facilities? (Probe interest, likes, dislikes, convenience)
- Timing- What is the preference/difference about receiving the reminders once per months, fortnightly, week before appointment day, two days to appointment day, etc? Why? Which is most helpful to you? In what way(s) is it more helpful?
- What would be the purpose of the reminders/voice calls/ messaging information or other mhealth technology?

D. Anxiety, technology engagement/fatigue

- When/how often would you need to receive the reminders/voice calls/information messages (or other suggested mHealth technologies); Probe; anxiety, fears to lose their other information by hitting a wrong button, fear of making mistakes.

- Generally, how do you find SMS/Audio messages if they were to be sent to you at scheduled time, say weekly, daily, bi-weekly as compared to your routine ones (probe bothersomeness, engagement, convenience, etc)

E. Self-efficacy, Behavioral intention to use

- How would you prefer the messaging program? (Prob needs for help, ability to read, understand messages, need for in-built help facility for assistance).
- Tell me about the last time you received any SMS that reminded you/encouraged you to do something important to you. What did you do immediately after receiving it? *Probe about the individual's particular experience. What was s/he doing? Where was s/he? How it transferred information intended. Probe for specific examples.*
- Has anyone ever told you they sent you an SMS or voice call that you didn't realize/see? Tell me what happened?/ why you think you did not receive the SMS/ voice call. *Probe about wrong phone number, phone malfunction/battery, migration, network connectivity, device failure (e.g., phone not working). etc.*
- Is there something about the way you use your phone that may have affected you getting the SMS/ call? What would you do to reduce these occurrences? (probe about strategies to network connectivity like double sim card phones, solar chargers, etc
- What concerns do you have about using mHealth technologies to support your ANC/hospital delivery utilization?, What would you change about the way SMS/audio calls are delivered? (Probe intention to use (& future use), recommendation to others
- What do you think about having your spouse or any member of your social network receive an mHealth intervention (eg. SMS reminders, voice call, messaging) regarding your ANC appointment or any other hospital appointment? What are the disadvantages

F. Social influence and facilitating conditions

- How do other people around you react when they see you read receive any call/ SMS? (probe about *social acceptance of the messaging/reminders/calls, probe spousal approval and support of the these, his experience and perceptions about them, motivation/ facilitators/barriers, resources to mobile use, compatibility with other phones)*

32. Is there anything else you would like to tell me about the other possible Mhealth technology approaches/improvements that could help you, act to remind or encourage you on your pregnancy journey that we haven't talked about?

Do you have any questions for me?

Thank you for your time.
