# Supplementary material for: mHealth-Based Health Promotion Intervention to Improve Use of Maternity Care Services Among Women in Rural Southwestern Uganda: Iterative Development Study
Source: JMIR Form Res. 2021 Nov 25;5(11):e29214. doi: 10.2196/29214 (PMC8663630; doi:10.2196/29214)
Supplement: Multimedia Appendix 2 [file formative_v5i11e29214_app2.pdf]

	Topic	Audio Messages - English	Audion Messages - Runyankole
1.	Introduction Audio	You are welcome to eBirth project which is implemented by Mbarara University of Science and Technology in Uganda. eBirth is a Patient-centered mobile technology interventionaimed at improving maternal health in Uganda. We shall provide you with regular pregnancy related SMS and or audio messages to offer you essential knowledge about antenatal care services, your health and that of your growing baby. We shall also provide you with upcoming clinic visit reminders to your phone. Some people from your social network may also receive these messages if you chose them to do so. Messages will be about topics related to pregnancy, labour and delivery, breast feeding, nutrition and postnatal care among others.<br><br>***These messages are not intended to replace your routine scheduled ANC visits. We hope you will find this useful for your health and that of your growing baby.***<br><br>***If you want me to repeat for you, press 1***<br><br>***Press 2, to continue***<br><br>***Press 3 to pause.***<br><br>***Press 4 to hang up….*****to repeat at the end of every message**	Nitukwaakiira omu Maternal Health Project etebekanisibwe Mbarara University of Science Entebekaniisa egyi neija kuba neshomesa abakazi abaine enda namaka gaabo ebikwatiriine nokureberera enda nabaana abarazaarwe turikusindika obutumwa ahasiimu. Nitwiija kuba nitukwohereteza obutumwa ahasimu yaawe. Obutumwa obwe nobwokukushomesa aha bikuru ahamagara gaawe waaba oine enda hamwe namgara gomwaana waawe. Ahasimu yaawe nainga eyoratuhe yoona, noija kuba noijusibwa ebiro byokureeba omushaho aheirwariro. Noobaasa kuterera omushaho waaba oine ekikushobeire. Obutumwa nibwija kuba nibukwaata ahamagara genda yaawe, okwetebekanisiza okuzaara, okwonsya, okuryagye nebiindi. Nitukwendeza okwejunisa entebekanisa egyi obase kuzaara omwaana wamagara marungi<br><br>Obutumwa obu tiburikukora omumwanya gwokuza kukyebeza enda yaawe omwirwariro, Kyebeza enda yaawe ori kukuratira abiro byabashaho barikukuha.<br><br>***Ku orabe noyenda nkugarikiremu nyiga emwe nana akadara.***<br>***Wabo otakyenda kurikiriza nyiga zero na akadara….*** **to repeat at the end of every message**
2.	**Pregnancy and role of ANC**	Today we are going to talk about signs and symptoms of pregnancy.<br>Here are some of the signs that can make you suspect that you are pregnant though many early pregnancy signs and symptoms can appear similar to routine pre-menstrual discomforts, tender, swollen breasts.<br>Note, your breasts may provide one of the first symptoms of pregnancy, followed by fatigue, slight bleeding or crumping, nausea without vomiting, food aversions and cravings, headaches, constipation, mood	Webaare kubanitwe omumushoomo ogu. Erizooba nituuza kuganaira ahabubonero bukwooreka ngu ogizire enda.Obu nibwo buumwe aha bubonero obwakubaasa kukumanyisa ku oine enda,nobu buraabe obumwe nibushushana nka waaba nooza kuza omu micwe y,ekikazi nka okuziimba amabeere.Ijuka ngu amabeere nigabaasa kuba akabonero k'okubaanza aha nda,hakurataho okubura amaani,okureeba akashagama kakye, kushaasha omunnda,kugira esheshemi otatanakire,okwaanga ebyokurya

		swings. This of course follow your missed periods. These are minor and will disappear with time. In case they become severe, seek medical advice. Whenever you miss your monthly periods after unprotected sex, you should suspect yourself to be pregnant. It's important for you to seek advice from a health worker to confirm pregnancy and start attending antenatal care. It's vital for you to attend antenatal in order to be helped, monitor the progress of your pregnancy with minimum interference; detect any deviation from normal at an early stage, be guided throughout pregnancy to have successful outcome, prepare you for labor and delivery. You also get treated for minor and major conditions that may come up during pregnancy.	bimwe,okugoomba,kuteerwa omutwe,kugoomererwa,n'okugira ekiniga maangu.Eki nikibaho wabura kuza omumicwe waaba otereine n'omushaija muteejuniise kapiira,kandi ebi nibimara obwiire bukye bimara bigyenda.Ku byakugumizamu,reeba omushaho.Ijuka kutaandika kucebeza enda kukihamya k'oyine enda kandi omanye enda yaawe oku eri n'okweri kukura.<br>Waaba noyeenda ngu nkugarukiremu,nyiga emwe.
3.	**Native medicine in pregnancy**	Today, we will talk about native medicine in pregnancy;<br>Native medicine means herbal medicine. During pregnancy, especially the first three months, the vital structures of your baby are being formed, so this process needs no interference.<br>Use of herbal medicine is common among pregnant women to treat nausea and vomiting, reduce the risk of pre-eclampsia, shorten labor, treatment of urinary tract infections and others.<br>However, these are dangerous to your health and pregnancy as their prescriptions and preparations is not proper with unclear doses. Some are for bathing, inhalation, chewing and seating in, others for insertion. Please restrain yourself from this for successful pregnancy outcomes. And for now thanks for listening, we meet next week.<br><br>***<u>Repeat after every other message:</u>***<br>***<u>These messages do not replace your routine ANC visits. Please attend your ANC as agreed with your HCP</u>***.<br><br>If you see the following, please seek medical advice immediately**.**	Eriizooba nituza kugamba aha mibazi y'ekiraguju waaba oine enda. Omu myeezi eshatu yokubaanza, ebicweeka ebikuru aha mwaana waawe nibiba birikuhaangwa.|Reero tikirikwenda biteganisibwe.Okukozesa emibazi egyo kiiri omubakyaara b'enda abingi. Bakugyezaho kuragurira esheshemi n'nokutanaka, kuzaara gye n'ebindi|Emibazi egyo, n'eyakabi aha magara gaawe n'enda yaawe. Entebekanisa yaayo n'ekipimo tibikukyeengwa.<br>Obyehara, kweenda otabaaruke gye.<br>Tubugane esande erikwiija.<br><br>***<u>Repeat after every other message or once weekly</u>***<br>Obutumwa obu tiburikukora omumwanya gwokuza kukyebeza enda yaawe omwirwariro, Kyebeza enda yaawe ori kukuratira abiro byabashaho barikukuha.<br>***Ku orarebe obubonero obu, oyirukangiire omwirwariro.***<br>***1. Okujwa***<br>***2. Pressure yaaza ahaiguuru***<br>***3. Omwaana atakutera***<br>***4. Omushweija, kuzimba ahamaisho nebigyere***<br>***5. Okuterwa omutwe gwamani***

		1. Vaginal bleeding<br>***2. High blood pressure***<br>***3.* severe headache, vision problems**<br>***4.* high fever, swollen hands/face**<br>***5.* Reduced fetal movement, or leaking amniotic fluid**	***6. Okwata eshaaho obwiire butakahikire***
4.	**Importance of early ANC's**	We hope you had a good week. We are back today, to talk about importance attending of antenatal care early. Whenever a woman suspects or confirms to be pregnant, its important that she starts her antenatal care early. This helps her to end up in good health and have a healthy baby too. Most women just attend antenatal care to get a card or know how her baby lies in the uterus, but it's important that she starts very early in order to know her Syphilis and HIV status, take folic acid early to prevent fetal complications, detect abnormalities at an early stage and be helped earlier, given health education on how to go about various issues and be helped to get successful pregnancy, labor, delivery and lactation. So be smart and keep in touch with your Health worker to be helped thoughout your pregnancy, review your progress and avoid complications.	Watekateka nanga okamanya ngu oine enda, nikirungi kutandika kugicebeza kare.| Eki nikikuyaamba kutabaaruka gye n'omwaana w'amagara marungi.|Abakyaara biingi nibacebeza kutunga ekipande ninga kumanya omwaana oku abyaami gye omunda|Kyebeza omanye waaba oine akakooko ka sirimu n'ebihooya,enda yaawe yaaba eine ekizibu ohweerwe kare kandi oshomesibwe.Guma hiihi n'omushaho akuhwere buri keire omurugyendo rwawe oyerinde akabi koona.
5.	**How babies grow in the uterus**	Today we are going to talk about how babies grow in the uterus;<br>Pregnancy is a good experience. Whenever a woman conceives, she is usually eager to know how her baby is growing. This helps her to be free with it and not taking it as a disease. You need information about your pregnancy, to know what happens at an early stage such that you may know how to go about it at every stage.<br>Fertilization happens when the sperms meets and penetrates an egg. Within about 3 days after conception the fertilized egg is dividing very fast into many cells. It passes through the fallopian tube into the uterine wall. The placenta, which will nourish the baby, also starts to form in the first three months, vital structures like the brain, heart, liver, kidneys, after then, the body forms	Omukazi weena ku arikugira enda,naaba aine ekihika ky'okumanya omwaana we oku ariyo naakura.eki nikimuyaamba obutagireeba nk'ekizibu ninga oburwiire|Omubiro bishatu otwiire,eihuri niritandika kwebaganisamu ebicweeka byingi,riraba omurushekye riza omuri nyin,enda|Ekyanyima,ebicweeka bikuru nk'ensigo, obwongo, omutima,nekine bikorwa.Nebicweeka ebindi bihangwa.Tayayira omushaho waawe kumanya omwaana okwarikukura

		other structures and keeps increasing in size and changing positions till it grows and stabilizes. To monitor this, you need to visit your midwife/doctor to help you know how your baby is growing at every stage on subsequent visits.<br>If you want me to repeat for you, press 1	
6.	**Keeping well during pregnancy**	We are now going to talk about keeping well during pregnancy;<br>Every woman once pregnant looks forward to having successful pregnancy/labor and postnatal period with a healthy baby. To achieve this you need to practice a few things like;<br>1. Drinking/eating a balanced diet in order to remain healthy.<br>2. Do regular exercises (not strenuous ones)<br>3. Avoid drinking alcohol<br>4. Ensure good personal of food hygiene<br>5. Avoid putting on high heeled shoes and tight clothes<br>6. Take a lot of fluids<br>Have enough rest by sleeping at least 8 hours in the night and 2 hors during the day.<br>Above all, attend regular ANC visit as scheduled by your midwife/doctor to get more information & get help always.<br>If you want me to repeat for you, press 1	Buri mukazi weena yagira enda, nateekateeka kutabaruka gye n'omwaana w'amagara marungi. Kuhikiriza eki,nootekwa kukora ebi:| 1. Okunywa munonga,n'okurya ebyokuryabyomugasho.<br>2. Kukora ebisasayizi byanguhi<br>3. Okwehara okunywa amaarwa.<br>4. Kuba omuyonjo.<br>5. Okwehara okujwara enkaito ningwa.| 6. Okuhumura eshaaha munaana nyekiro, n'eshaaha ibiri omwihangwe.<br>Okukira byoona, n'okucebeza enda nk'oku omushaho yakugambira burikeire otakwosha.
7.	**Minor disorder of pregnancy**	Today we shall talk about minor disorders of pregnancy.<br>Every pregnancy is a unique experience for women, with different pregnancies coming with different feelings. You may experience common disorders such as nausea, vomiting, loss of appetite, dizziness, backache, leg crumps and constipation. You are advised to visit your health worker for proper evaluation as most of these conditions are treatable, to achieve desired pregnancy outcomes for you, your baby and the community.<br>If you want me to repeat for you, press 1<br>Press 2, to continue	Burinda eine emitwaarize yaayo aha mukazi kandi tizirikushushana. Omukazi w'enda nabaasa obutabeerwa gye,nk,okugira esheshemi,okutanaka,|okuremwa kurya,okushaasha omugoongo n'amaguru,okugoomererwa ,n'oruzengyerera.noobaasa kubitunga ninga obutabitunga.|Kwonka kuwa kugira bimwe omuri ebyo ninga byoona,tayayira omushaho waawe ah'irwariro ryoona eryorikwenda akuyambe.Eki nikija kukuyamba kutabaruka gye.

		Press 3 to pause.<br><br>Press 4 to hang on.	
8.	Bleeding in pregnancy<br><br>(APH)	Let's talk about bleeding in pregnancy;<br><br>When a woman becomes pregnant, bleeding stops. For any bleeding that happens thereafter is abnormal. In early pregnancy, this could mean youmay have an ectopic pregnancy or abortion process but in pregnancies after 7 months, it means there is a big problem with the placenta. Bleeding can cause the death of your baby in the uterus and leave your life in danger. Ensure you receive proper examination by your midwife. Always take your iron tablets every day to keep good levels of blood. Have a balanced diet to keep healthy<br><br>If you want me to repeat for you, press 1	Omukazi kwaaba ayine enda tareeba eshagama. Okujwa kwoona oine enda ninga noteekateka kuba oine enda tikiri kirungi. Okujweera enda ekiri nto nikibaasa kumanyisa ngu enda eine ekizibu nkenda kurugamu. Okujwa ahameezi mushanju nokugaruka aheiguru nikimanyisa ngu ekyanyma kyenda yaawe kiine ekizibu kihaango munoonga. Okujwa nikubaasa kwiitira omwaana omunda kandi kute amagara gaawe omukabi kahango. Reeba ngu omuzarisa yakyebera gye enda yaawe, okore scan omanye ekyanyima kyenda yaawe okukyemereire. Mira obujuma bweshagama burizooba kureka omutiindo gweshagama yaawe aheiguru. Rya ebyokurya ebyomugasho oba balanced diet burizooba ogire amagara marungi.
9.	Male partner involvement	Today we shall talk about male partner involvement; The father of your unborn baby is as important as you the mother in the care of your pregnancy. You are encouraged to involve him in the affairs of this pregnancy. Encourage him to accompany you to the health facility during all scheduled antenatal and postnatal visits where you will both be tested for HIV and Syphilis, know your blood groups and receive important health education messages together, to help you deliver a healthy baby. Your partner will support you best if he is involved from the beginning. Discuss with him about your birth plan and ensure the funds to facilitate the delivery process are available. Remember you may develop complications requiring referral to another health facility. Prepare for such emergencies all the time.<br><br>If you want me to repeat for you, press 1	Eirizoba nituzakuganiira aha buvunanizibwa bwomujaija waawe. Ishe womwana wawe owoyiine omunda ayiine omugasho ogurikwingana nogwawe nyina womwana. Nohigwa kukwatanisa nawe omubwire bwenda yawe. Omuhigye kukushendekyereza ahirwariro emirundi yoona eyiwaragyirwe kureeba omushaho reru mukyebeze hamwe akakoko kasirimu hamwe na syphillis, omanye omuringo gweshagamaye kandi mutungye hamwe okwegyesibwa kwomutano ahabyamagara Ekyo nikyiza kukuhwera okuzara omwana wamagara marungi. Omukundwa wawe naija kukuhwera kurungi waheza kumwehisya.

10.	ANC Visit schedules & Planning	Let's now talk about ANC visit schedules;<br><br>Always plan to attend all scheduled ANC visits with a Health worker. Ministry of Health recommends a total of at least 8 visits, once every month. Start as soon as you miss the second consecutive menstrual period. You may return for early check up whenever you have any danger sign like nausea and vomiting, cravings, constipation, bleeding from your private parts. Ensure the health worker provides all recommended services at each visit including TT injections, Malarial prophylaxis, physical examination, checking your weight and blood pressure at every visit, laboratory tests like HIV and syphilis, urine analysis, Hepatitis B, Haemoglobin estimation. Ask for mosquito net on first visit. In the last visit, discuss your delivery plans with the health worker. Ensure your husband has enough information to be able to support you.<br><br>If you want me to repeat for you, press 1	Eirizoba nituzakuganiira aha kuchwebeza enda.<br><br>Rebeka ngu wayetebekanisa kandi watayayira omushaho otendekirwe emirundi yoona eyimwikiiriziineho ahabw'okureberera gye endayawe. Minisiture yebyamagara neragiira okutayayiira omushaho emirundi etari hansi yamunana, omurundi gumwe burikwezi. Rebeka ngu omushaho yakuhereza obuhereza bwoona oburagiirwe buri kwija kuchebeza. Obu burimu obukatu bwa TT, okukyebera omushwija gwensiri, okukyebera endebeka yaawe, okupiima oburemezi bwawe, okupiima pressure, okukyebera akakoko kasirimu hamwe nebihooya, okukyebera enkari, hepatitis B hamwe nokupiima obwingyi bweshagama. Rebeka ngu watunga akatiimba kensiri wiija kuchebeza omurundi gwo kubanza kandi ogarukye owomushaho burikeire ku orahurire oburemezi bwoona.
11.	HCP interaction	Confirming your pregnancy and your baby's progress empowers you to make the right decisions. Report to a qualified health care worker as soon as you feel unwell. For individuals with complications or chronic conditions like diabetes, hypertension, it's good to always interact and discuss pregnancy intentions or progress with a doctor in case medications need to be changed to suit pregnancy. Know early, Be smart and live a healthy life for you and your baby.<br>If you want me to repeat for you, press 1	Eirizoba nituzakuganaira ahakuganiira nomushaho waawe waaba oine enda. Okuhamya kwoyiine enda narishi oku omwana waawe arikukura nikyikwongera amaani okukora enchwamu eboneire. Oshemerire kureba omushaho aho naho waba otakwehurira nkaburizo. Aharyabo abiine oburemezi nko bwo burwire bwa shukari, pressure, nikyirungi kuganiira ahabyenda yawe nomushaho kugira ngu haaba nihetengwa okuhindura emibazi, obaase kuheebwa emibazi eshemerire enda yawe. Manya kare oyetebekanise kuzaara omwaana wamagara marungi.
12.	Nutrition	Like any other person, a pregnant and breastfeeding woman needs to eat a balanced diet at all time to have a healthy baby and she also remains healthy. A	Eirizoba nituzakuganaira aha kurya kwaawe. Gumizamu orikurya ebyokurya byemiringo mirungi.-kwenda ngu oyije ozare omwana woburemezi buringanire- kwenda ngu otungye

		balanced diet includes carbohydrates, fats, proteins and minerals/micronutrients.<br>Remember: pregnancy increases all nutritional requirements, hence the need for special attention. Ensure an extra meal from the usual routine, take daily folic acid and iron tablets, deworming using mebendazole. Take iron and folicrich foods like green leafy vegetables, meat, millet.<br>If you want me to repeat for you, press 1	ebyokurya ebirikwombeka omubiri,, ebyokurya ebirikuha amaani, ebishaju hamwe nebirisa ebindi ebirikwetengwa omu kukuza omwana.-kwenda ngu otungye eshagama hamwe norwizi rwinji omumubiri kuyamba omukubobeza omwana ariyo nakura omunda.-kwongera okukuza ekyanyima kandi nokukyongyera kukora gye.-kutunga ebirisa ebirikumara ebirimu folic acid hamwe nebindi nka zinc, na iodine.-kutunga vitamins zirikumara ezirimu A,B,C, D kukuma amagara marungi gomukazi hamwe nomwana owomunda.-kuyamaba kwongera calcium erikwetagwa omu kukora enyama na magufa gomwana.
	Nutrition 2	Continue taking a balanced diet<br>• To attain an average baby's weight at birth<br>• To have extra proteins, carbohydrates, fats and other nutrients needed for the baby's growth<br>• To build extra blood and body fluids for nourishing the growing baby<br>• To facilitate placenta growth and optimum functioning<br>• To get sufficient minerals like Iron and Folic acid plus other micronutrients like Zinc, Iodine,<br>• To have enough Vitamins like A, B series, C, D to maintain a woman's good health and the health of the growing baby<br>• To increase calcium intake that is required for formation of baby bones and muscles	
13.	Danger signs during pregnancy	Pregnancy may come with some undesirable effects like nausea and vomiting, cravings, constipation, bleeding from your private parts. Report to the health work for advice in case you experience any disturbance during this period. Ensure to have an early pregnancy ultra sound scan to exclude twins and ectopic pregnancy. Bleeding and abnormal discharge may be a sign that your pregnancy is experiencing challenges. Seek	Eirizoba nituzakuganaira ahaobubonero bwakabi ahanda.<br>Enda nebasa kwija nobubonero bwotarikukunda oburimu esheshemi, okutanaka, okugomba, okugumirwa omukushohoza hamwe okurigwamu eshagama omubichweka byawe byekyihama. Ogambire omushaho otendekyirwe waheza kuteganisibwamu. Rebeka ngu waza omuscan endayawe ekyirinto kwenda ngu omanye yaba oyiine abarongo ningashi enda yaba etashutamigye omuri nyinenda. Watandiika

		medical attention as soon as you notice any changes.<br>If you see the following, please seek medical advice immediately**.**<br>***1. Vaginal bleeding***<br>***2. High blood pressure***<br>***3.* severe headache, vision problems**<br>***4.* high fever, swollen hands/face**<br>***5.* Reduced fetal movement, or leaking amniotic fluid**	kurugwamu eshagama narishi amaizi gataboneire nakamanyiso ngu endayawe eyiinemu oburemezi. Reba omushaho ahonaho waheza kureba empinduka.
14.	HIV and syphilis testing for mother and Partner	At your first ANC visit, ensure your health worker tests your blood for HIV. You will then be asked to repeat HIV testing very three months until end of breastfeeding. HIV infection can be transmitted to the unborn baby or during delivery and breastfeeding period. HIV infection can give poor quality of life to your infant. In case you are infected, do what it takes to prevent transmission to your baby. Health workers have effective preventive measures including ARVs. Know your status to be able to save your baby	Eirizoba nituza kuganiira aha kukyebeza oburweire bwa siriimu n' ebihooya. Ahamurundi gwawe gwokubanza kukyebeza enda, rebeka ngu omushaho yakukyebera eshagama kureeba akakoko kasirimu n'ebihooya. Aho noija kushabwa kugarukamu kukyebeza buri myezi eshatu mpaka ahamuheru gwokwonsya. Akakoko kasirimu nikabasa kuturizibwa omwana atakazirwe ningashi omubwire bwokuzara n'kwonsya. Akakoko kasirimu nikabasa kureta amagara mabi aha mwanawe wawe. Kyabaho okaba okiine, kora kyona ekyirikwetagisa obutakaturiza omwanawawe. Abashaho beine emiringo eyokubasa kutangiramu erimu ARVs. Manya oku oyemeriire obase okujuna omwanawawe.
15.	Prevention of Malaria in pregnancy	During pregnancy you need to prevent and control malaria through sleeping under insecticide treated mosquito net, ensuring that you destroy all breeding sites for mosquitoes-clean the compound and keep it slashed, remove empty tins from the compound, make sure there is no stagnant water in the compound and close the windows of your house early. Ensure that you receive fansidar at each ANC visit. Seek medical care promptly whenever you develop fever. Do not take unnecessary medications in pregnancy because some may harm your developing baby especially in the first 3 months<br>If you want me to repeat for you, press 1	Eirizoba nituzakuganaira ahakwetantara omushwija gw'esiiri.<br>Omubwiire bwenda yawe, noyetenga kurwanisa kandi nokuzibira omushwija gw'ensiri obwo orikubyama omukatiimba kensiri akafuhirirwe. Rebeka ngu wamaraho emyanya yona eyensiri zirikuzariramu, shuusha embuga yawe kandi oreebe ngu yaguma eri enyonjo, otorotore ebikyebe byona kuruga omumbuga yawe, orebekye ngu tihateramamu amaizi mabi kandi okyinge amadirisa genju yawe bukyirikare.Tunga fansidar buri murundi gw'oratayayire omushaho. Roonda obujanjabi ahonaaho wahurira omushwija. Otakyigyeza okamira emibazi etakwatirine nenda, ahabwokuba nebaasa kushiisha omwana wawe ow'omunda namunonga omumyeezi ashatu y'okubanza.

16.	Care of HIV in Pregnancy	HIV can be transmitted from an infected mother to her baby. Know your HIV status at the first antenatal visit and repeat the test every 3 months until end of breast feeding. For those who test negative, ensure to remain HIV negative to keep healthy. If you are found HIV positive, ensure to receive appropriate care from your antenatal clinic and ART clinic. You will receive ARVs daily for life to protect your health and prevent transmission of HIV to your baby. Ensure to deliver in the hospital where your baby will receive ARV syrup daily for 6 to 12 weeks. Your baby will be followed up in an exposed infant's clinic where HIV tests for the baby will be done at 6-10 weeks and repeated at 6 weeks after breast feeding. Do all it takes, receive all the recommended care to have an HIV free baby	Eirizoba nituzakuganaira aha kwekuma gye waaba oine siriimu obwiire bwenda. Sirimu nebaasa kuturira omwaana kuruga ahari nyina yaaba ayine silimu. Manya embeera yaawe, okyebeze akakooko ka silimu waija kukyebeza enda omurundi gwokubaanza, garuka okyebeze buri myeezi eshatu kuhisya ah'orahereze kwonsya. Ku arakyebeze okashanga oteine kakooko ka siliimu, kora kyoona akirikubasika okuratire obuhabuzi bwabashaho kuguma oteine akakooko aka, ogume oine amagara marungi. Ku orashange okiine, reeba ngu waheebwa obuheereza bwomutiindo ahi orikuckyebereza enda ninga omu clinic ya siliimu ahirwariro. Nibiija kukuhereza emibazi ya ARVs burizooba kukyingira amagara gaawe kuza omukabi, reero obaase kutaturira omwaana waawe akakooko ka siliimu. Zaarira omwirwariro buriijo ahu omwaana aha'ratungire obuhereza oburimu ARV syrup buriizooba kumara esande 6 kuhisya 12. Abashaho nibiija kuguma nibareberera omwaana ogu omu clinic yabaana abu ba nyina biine akakooko ka siliimu. Omwaana naija kukyeberwa nokukakasa ku ahonire sliimu ahasande 6-10, agarukye ahamizibwe esande 6 acuukire. Kora kyoona ekirikubasika, tunga obujanjaabi obuhamiziibwe abase kuzaara omwaana otiine sillimu.
17.	**Travelling during pregnancy**	Whenever you are pregnant, there is no need to worry about travelling, as long as there are no identified complications or concerns with your pregnancy. The best time to travel during pregnancy is the second trimester because in most cases you are passed the morning sickness of the first trimester and several weeks from the third trimester when you're more easily fatigued. The risk of travelling long distances is that; it increases the risk of clots forming in deep veins of the leg, known as deep vein thrombosis (DVT). The risk is increased in pregnancy if you had a DVT in the past.<br>If you want me to repeat for you, press 1	Erizooba nituza kugamba aha kutambura omu safari waaba oine enda. Buri waaba oine enda,tikirikukuzibira kutambura orugyeendo ruriingwa enda yaawe yaaba eteine buzibu.Obwiire burungi bw'okutamburiramu nahagati y'emyeezi ena kuhika ah myeezi mukaaga ahakuba omu bwire obu,nooba otakibura maani nk'omumyeezi y'okubaanza ,kaandi nihaba nihaburayo emyeezi y'okukurusya.Akabi k'okutambura engyeendo niingwa nokugira ngu nikireeta eshagama yayekwaata omu misi yamaguru ku orikuba waragizireho ekizibu eki.

18.	Birth preparedness	Good pregnancy outcomes depend on the level of preparation. Be well prepared for the birth of your baby by discussing your plans with your main caretaker and health worker. Be sure of the name and location of the health facility you wish to deliver from. Arrange transport means to your preferred health facility. Be ready for emergency referral in case its necessary. Ask your midwife about items to bring for delivery like maama kit. Have enough personal items like clean clothes, pads, basin and baby clothes<br>If you want me to repeat for you, press 1	Eirizoba nituzakuganaira aha kwetebekanisiza okuzara.<br>Okutunga ebirungi okuruga omunda eyoyiine nikyirugirira okwoyetebekanise. Rebeka ngu wayetebekanisa gye okuzara omwanawawe, orikuganiraho n'omushaija wawe hamwe nomushaho wawe. Rebeka ngu wayetegyerezagye eiziina n'omwanya gwirwariro eryorikwenda kwija kuzariramu. Tebekanisa kurungi akasente akarakuyambe kukuhitsya omw'irwariro. Rebeka ngu oyetekateekyire kwongyerwayo omw'irwariro erindi ahonaho kyayetagisa. Buuza omuzarisa wawe ebintu ebi oshemereire kugyenda nabyo waba noza kuzaara ebirimu maama kit. Oshemereire kuba oyine ebikwato ebyawe nkomuntu birikumara ebirimu emyenda etebekanisibwegye, pads, ebafu hamwe nemyenda yomwana.
19.	Planning Birth	You are now in the last months of your pregnancy journey! Do you feel you are well prepared for the birth of your baby? Which health facility will you go to for delivery when labour begins? Who will accompany in labour, what means of transport will you use in emergency situation. Have you saved enough money to use during this period? Do you have anyone to care for your home and other children when you go for delivery? Know from your midwife if there are some supplies you may need to come with including gloves, akaveera, syringes. Have your maama kit with you all the time in case of an emergency. Prepare enough clean clothes for your use while in the hospital. ensure you have enough clothes for the baby including gloves and caps.<br>If you want me to repeat for you, press 1	Eirizoba nituzakuganaira aha ntebekanisa yokuzaara.<br>Hati wahika aha myezi yokushembayo omurugyendo rw'enda yawe! Nohurira oyetebekanise kurungi okuzarwa kwo omwana wawe? Noza kuza mw'irwariro ki kuzara ebisha kubiratandike? Nooha arakushendekyereze waba ori aha bisha, noza kukozesa ntamburaki omubwetago bwahonaho. Obikyire esente ezikumara ezokweyambisa omubwire obwe? Oyine omuntu arakuyambe nk' okureberera ekayaawe, hamwe nabaana abandi waba ogiire kuzaara? Manya okuruga ahamuzarisa waawe kuharaabe hariho ebyorikwetenga ebirimu gloves, akaveera, n'eboomba. Guma oyine maama kit yawe obwire bwoona nihabasa okubaho obwetagyi bwahonaho. Tebekanisa emyenda eyogize erikumara eyorayeyambise waba ori omwirwariro. Rebeka ngu oyine emyenda yomwana erikumara erimu gloves n'engofire.
20.	Facility delivery	As you know you are coming closer to the day of delivery of your baby. It must be exciting but also full of many uncertainties. The ultimate goal is to deliver a healthy baby and you also remain strong enough to continue offering your baby recommended care. Every mother must discuss with the	Eirizoba nituzakuganaira aha miringo yokuzaara. Ekiro kyokuzaara omwaana waawe kiriyo nikirira haihi. Kishemeriire kukuta akamweenyo aheitama kwonka ebingi torikumanya okubiraagyende. Ekigyedererwa

		midwife the mode of delivery of her expected baby. We always recommend every woman to deliver from a health facility. Remember a natural birth is the best once supervised by qualified health worker. For those unable to have a normal delivery, operations an option. Whenever you need an operation, ensure you get it timely. It's a lifesaving procedure for the mother and the baby. Plan to deliver in a health facility able to offer an operation in case you need it or be prepare for urgent transfer to a facility able to do an operation without delay. If you have been operated before, you may require another operation before labour begins. Be sure of the mode of delivery of your baby when you are still pregnant to make the correct decisions before costly complications set in<br>If you want me to repeat for you, press 1	ekikuru nokuzaara omwaana wamagara maruungi neiwe okaguma oine amaani gokumureberera okukishemerire. Buri mukazi ashemereire kugaanira nomuzaarisa oku arizaara omwaana, okuzaara gye ninga okushamezibwa. Abashaho nibahabura buri mukyaara okuzaarira omwirwariro burijo. Ijuka ngu okuzaaragye nikyo kirungyi obwe waaba ozaarire omwurwariro omubashaho abatendekirwe gye. Ahari abo abatarikubaasa kuzaara gye ahabenshonga nyiingyi, okushemezibwa nikwiija kugarura amagara gaanyu ahamutiindo. omukyaara weena otarikubaasa kuzaara gye ashemeriire kushemezibwa embbera zitakahindukire zikata magara ge nagomwaana omukabi. Tebekanisa kuzaarira omwirwaariro aryakubaasa kukushemeza kukirayetaagwe nainga ogume nentebekanisa yokwirukangibwa omwirwariro erihango ahonaho. Ku araabe arashemeiibweho, nobaasa kwewtenga kushemezibwa ebisha bitakatandikire. Manya okworaazaare omwaana waawe okiine enda, asharemu kuruungi atatiire amagara gaawe nomwaana omukabi.
21.	**Importance of being delivered by a skilled proffessional**	Midwives play an instrumental role to introduce women to the health system and ensure that women and their babies receive a continuum of skilled care during pregnancy, child birth and in the important days and weeks after birth. You need to be close to your midwife in order to help you go through pregnancy, labor, and deliver successfully.<br>Many times, midwives have been labeled being rude to mothers and women end up going to unskilled birth attendants and most	Erizooba nituza kugamba ahaAbazaarisa baine omwooga muhaango gw'okuhikanisa abakyaara n'abaana obuheereza bw'amagara ,kureeba ngu abakazi barebererwa gye baine enda,omukuzaara,na baheza kuzaara.Oine okuba hiihi n'omushaho waawe kikuyaambe kutabaaruka gye.Kaingi abazaarisa nibamanywa ahabw'okugambira kubi abakyaara,baheendera bagiire omubazaarisa batatendekirwe bagirirayo ebizibu.N'obugabe bwaawe kunywaana,okaroonda omuzaarisa orakuyambe omukuzaara ahabwokuba nooba

		of them get complications which are irreversible by the time they come to hospital. Its your right to befriend and choose midwife to deliver you since you have built that relationship during the subsequent visits of ANC so try to be open, friendly, close to midwives and use them to get you well for labor and delivery.<br>If you want me to repeat for you, press 1	wamumanyiire orikucebeza enda.Nahabw’ekyo gyezaho kwehisya,onywaane abazaariisa ,bakuyambe kutaabaruka gye.<br>Ku orabe noyenda nkugarikiremu nyiga emwe
22.	Care during labour	When labour pains begin you begin a new experience. When you are well prepared you will enjoy the experience. Report to your planned health facility for delivery for initial assessment. The midwife will monitor the progress of labour by repeated examination for you and the baby till delivery. Ensure proper hygiene in labour by bathing regularly and use clean sanitary towels. You are advised to have small frequent nutritious meals and teas to keep health and well hydrated. Report any undesirable occurrences at any time. A good midwife will prepare you for pushing your baby when delivery time comes. After delivery your baby will receive immediate care to help initiate breathing and treat any difficulties. The midwife will ensure you are not bleeding by giving you an injection. The baby will be weighed and immunized before discharge. Deliver under qualified health workers to give your baby a good start<br>If you want me to repeat for you, press 1	Eirizoba nituzakuganaira aha kuraba gye omubwiire byokuzaara.<br>Ebisha kubirikutandika, oyeshanga omukintu ekitari kya buriijo. Ku orikuba oyetebekaniisize gye, noija kubishemerererwamu, ayanguhirwe, ogume nobusingye hamwe naabo abariniiwe. Gyenda ahirwariro eri otebekanisize kuzariramu bakyebere bamanye embeera yaawe n’omwaana. Omuzaarisa naija kukuguma haihi, obwe arikukukyebera iwe nomwaana waawe kuhika ahi arazaarire. Ba omuyoonjo, onaabe burikaanya omubirii gwoona kaandi ojwaare paadi. Ogume noorya ebyokurya n’ebyokunywa ebirimu ekiriisa ekirikumara ogume oine amaani kaandi otahwamu amaizi omumubiri. Manyisa omuzaarisa byoona ebirakuhikeho ebi atarikukyeenga. Omuzaarisa murungi naija kukutebekanisa gye oku orasindike omwaana waawe eshaaha yahika. Waheza kuzaara, omwaana naija kuheebwa obujanjaabi kumuhweera omukwiisya nokukumaraho ebindi bizibu byoona. Omuzaarisa naija kukuteera akikatu kyokukuzibira kujwa, apime oburemeezi bwomwaana, amugyeme otakatahire. Zaarira omubashaho abatendekirwe ohe omwaana waawe entandikwa enuungi.<br>Ku orabe noyenda nkugarikiremu nyiga emwe

23.	Family Planning	Family planning means having children by choice and not by chance. It's the single most effective means of preventing maternal morbidity and mortality. Let every mother have a child when she is ready, and avoid finding yourself pregnant even when you do not feel you want to. Receive family planning information during pregnancy. The majority of women will return to normal fertility by 2 to 6 months following the birth of their baby. You do not have to first experience your monthly period in order for you to become pregnant. Ensure that you are using a family planning method by 2 months after delivery for be on a safe side. NO WOMAN SHOULD LOSE LIFE WHILE GIVING BIRTH.<br>If you want me to repeat for you, press 1.	Eirizoba nituzakuganaira aha Okubaririra oruzaaro. Okubaririra oruzaaro ninga family planning nokuzaara abaana aborikwenda, aboshaziremukuzaara. Family planning tikirikumanyisa okuzaara abaana fwaa, niinga aha chance. Ogu nigwe omuringo gumwe ogurikutambira abakyaara beenda kufa nibazaara. Nitushaba burimukyaara kuzaara omwaana owu ayetekatekyiire, reke kweshanga oine enda eyi otarikweenda. Tuunga obuherezan'okushomesibwa ebya Family Planning okiine enda. Abakyaara abingi nibabaasa kugaruka kugira enda ahameezi nkaabiri kuhisya ahameezi mukaaga baherize kuzaara, baaba niboonsya ninga batari kwoonsya. Toshemeriire kutegyereza okugaruka omukweezi kugira enda, reka omwaana abanze akure. Reebeka ngu ori ahamuringo gwa family planning omwaana akyiine ameezi abiri oherize kuzaara okweeha obusiingye, oshemeze amukago gwaawe nomukundwa waawe. TIHARIHO OMUKAZI OSHEMERIIRE KUFA ARIKUZAARA
24.	Immunization & caring for the baby	It's good for every mother to know whether her baby is growing well. You are encouraged to breastfeed your child exclusively as soon as it is born. At discharge, the health worker will give you return dates for immunization when the baby will be weighed to determine the development of your baby. Your baby will be weighed at birth and 6, 10 and 14 weeks whenever the child receives immunization. Continue bringing the baby for at 6 months when the baby will also receive Vitamin A and at 9 months during measles immunization. When your baby is found with abnormal growth, you will be advised among other things on how best to feed your bay for proper development<br>If you want me to repeat for you, press 1	Nikirungi buri muzeire kurebereragye omwanaawe akamureeba nakurakurana kurungi. Nitukushaba kwonstya omwana waawe otamuhire kintu ekindi kyona kumara ameezi mukaaga agokubanza. Waheza kuzara nohebwa obunjanjabi oburimu okutangira hamwe nokuragurira oburemezi. Omuzaire nahwerwa okutandika okwonsya omushaha emwe eyokuzara. Rebeka ngu omuzarisa wawe yakuberaho omushaha mukaga ezokubanza okwenda kukuma amagara marungi. Nyaburawe garuka kureeba omushaho bwanyima y'esande mukaga kwenda kureeba okwoyemerire hamwe nomwana wawe, nokugyemesibwa. Okutayaya okundi nikuba nikuzakugaruka kubaho bwanyima y'esande 10, 14 ah'omwanawawe egyemesiibwe kandi niwe otungye amakuru gokubarira oruzaro hamwe nenkura yomwana waawe. Ameezi mwenda kugyemesibwa obusheru nanokukuyamba ahankurakurana yomwana waawe.

25.	**Dangers of grand multi parity**	It is usually good for a woman to deliver few children which she can manage and reduce on pregnancy related risks. Grand multiparity remains a risk in pregnancy and associated with increased maternal and neonatal complications like, malpresentation, placenta previa, low Apgar score in new borns, meconium stains liquor etc which puts your life and baby at stake. Being a grand multipara is a high risk because the adverse outcomes can head to social economic burdens to the mother/family and health systems. So it is very important for you to discuss with your health worker concerning birth control.<br>If you want me to repeat for you, press 1	Erizooba nituza kugamba aha kuzaara abaana baingi.Nikirungi omukazi kuzaara abaana bakye abarikubaasa, kweerinda akabi aha magara ge .Okuzaara abaaana biingi n'ekyakabi aha mukazi n'omwaana ,kireetera omwaana kubyaama kubi omunda,ekyaanyima kubandiza omwaana,akazaarwa aruhire,kugira eshoha etabonire hamwe n'ebindi ebyaakubaasa kuta amagarag'omwaana omukabi.Okuzaara munonga nikireetera omukazi amagara mabi'obutashemererwa hamwe n'obwooro omuka.Nahabw'ekyo,nikirungi okugaaniraho n'omushaho waawe ahabikwatiriine n'embariirira y'oruzaaro.Webare kunpurikiriza.<br>Waaba noyenda nkugarukiremu, nyiiga emwe.
26.	Post Natal Danger signs	Today, we will talk about some of the danger/warning signs that you need to look out for after childbirth. Heavy bleeding, swollen eyes, fever, chills, vomiting, chest pain, trouble breathing, severe headache and extreme pain in legs, stomach, back, lower abdomen or pelvis, foul smell, blocked or painful breasts, jaundice (baby), excessive bloating, paleness, loss of appetite and general malaise. Watch out and whenever you see these signs, please seek medical attention quickly.<br><br>If you want me to repeat for you, press 1	Eirizoba nituzakuganaira aha bubonero bwakabi waheza kuzaara.<br>Ku oratungye bumwe aha bubonero obu omubwire obwo oherize kuzara omwana wawe, garuka omwirwariro ahonaho. Burimu okujwa munonga, okuhaga ahameisho, omushweija, okutanaka, okurugamu amaizi garikunuka kubi omubichweka byekihama, obusaasi bukyebukye omumaguru, omubiri gwa kinekye ahamwana, kubura kurya, obusaasi hona hona, okushasha omunda butosha, okwijura enda hamwe nokuburwa amaani omu mubiri gwona.<br>Obutumwa obu tiburikukora omumwanya gwokuza kukyebeza enda yaawe omwirwariro, Kyebeza enda yaawe ori kukuratira abiro byabashaho barikukuha.
	Default SMS Reminder to social supporters	We appreciate your being consistently close to your friend XX who is pregnant, we are reminding you of her upcoming antenatal visits on the date indicated on her card.	Webaare kuguma haihi namunywani waawe oine enda. Nitukwijutsya ku aine okuza ahairwariro kucebezaenda aha kiiro ekihandikiire aha kadi yaawe.
